# Favorable short-term survival in locally advanced cervical cancer with optimal pathological response after neoadjuvant chemoimmunotherapy: a real-world analysis

**DOI:** 10.3389/fimmu.2026.1737081

**Published:** 2026-04-07

**Authors:** Luyang Zhao, Qingzhi Zhai, Wenbo Zhang, Liang Wen, Xiaoyu Fu, Xiufeng Xie, Xiangshu Jin, Nina Zhang, Yuanguang Meng, Li’an Li, Chenglei Gu

**Affiliations:** Senior Department of Obstetrics and Gynaecology, The Chinese PLA General Hospital, Beijing, China

**Keywords:** locally advanced cervical cancer, major pathological response, neoadjuvant chemoimmunotherapy, pathological complete response, survival outcome

## Abstract

**Background:**

This real-world study evaluated short-term survival outcomes and potential risk factors in patients with locally advanced cervical cancer (LACC) who achieved optimal pathological response (OPR) following neoadjuvant chemoimmunotherapy and radical surgery.

**Methods:**

A retrospective analysis was conducted on LACC patients treated at a high-volume tertiary medical system between 2022 and 2025. All eligible patients received neoadjuvant chemoimmunotherapy followed by radical hysterectomy. Pathological response was categorized as pathological complete response (pCR; no residual tumor) or major pathological response (MPR; ≤10% residual tumor). Clinical data, treatment details, and follow-up information were systematically collected. Additionally, transcriptomic profiling and multi-algorithm immune infiltration consensus analyses were performed on matched pre- and post-treatment tumor specimens to uncover treatment-induced microenvironmental remodeling.

**Results:**

Among 89 eligible patients who underwent surgery, 32 (35.9%) achieved an OPR, comprising 18 (20.2%) with pCR and 14 (15.7%) with MPR. Over a median follow-up of 13 months, the estimated 2-year disease-free survival was 90.7% with an estimated overall survival of 100%. No specific baseline clinicopathological factor emerged as a significant predictor of recurrence in univariable analysis. The regimen was well-tolerated, with grade 3 treatment-related adverse events occurring in 21.9% and no grade 4–5 events reported. Transcriptomic profiling of 10 paired pCR specimens revealed preliminary microenvironmental remodeling post-treatment, characterized by the activation of the NFAT signaling pathway and extracellular matrix reorganization.

**Conclusion:**

LACC patients attaining pCR or MPR after neoadjuvant chemoimmunotherapy and surgery demonstrated excellent short-term survival outcomes. These findings provide a rationale for considering de-escalated adjuvant therapy in this highly responsive subgroup. Validation through larger prospective cohorts with extended follow-up is warranted.

## Introduction

Cervical cancer ranks as the fourth most common and lethal malignancy among women worldwide, with Chinese accounting for approximately 23% of the global burden (1). While early-stage cervical cancer is associated with favorable outcomes, boasting 5-year overall survival (OS) rates exceeding 90% (2), locally advanced cervical cancer (LACC) presents a considerably poorer prognosis, with reported 5-year disease-free survival (DFS) and OS rates of 68% and 74%, respectively (3). Globally, 37% of cervical cancer cases are diagnosed at the locally advanced stage, a proportion that exceeds 50% in developing countries (4), representing a persistent threat to women’s health.

Concurrent chemoradiotherapy (CCRT) remains the standard first-line treatment for LACC (5). However, significant disparities in healthcare resources and infrastructure across regions hinder its universal adoption. In China, for instance, a nationwide physician survey revealed that CCRT alone is utilized in only 18.1%, 42.2%, and 52.1% of patients with stage IB3–IIA2, IIB, and III–IVA disease, respectively. Conversely, surgery-based multimodal approaches are employed in 67.1%, 42.0%, and 26.6% of cases across these corresponding stages, underscoring the pressing need for effective alternative strategies (6).

Historically, neoadjuvant chemotherapy alone provided marginal survival advantages over definitive CCRT (7). However, the recent integration of immune checkpoint inhibitors (ICIs) has revolutionized this paradigm. Landmark prospective trials, including the NACI, PACS, and NATIC studies, have demonstrated that combining ICIs with neoadjuvant chemotherapy (neoadjuvant chemoimmunotherapy) yields unprecedented pathological complete response (pCR) rates ranging from 33.3% to 66.7%, alongside manageable toxicity profiles (8–10). Despite these striking pathological outcomes, the long-term prognostic significance and the precise molecular mechanisms governing optimal pathological responses (OPR: pCR or major pathological response [MPR]) in a real-world setting remain incompletely elucidated.

Therefore, this study aimed to retrospectively evaluate the short-term survival outcomes of LACC patients who achieved OPR following neoadjuvant chemoimmunotherapy. Furthermore, by integrating transcriptomic sequencing and multi-algorithm immune consensus, we sought to delineate the microenvironmental dynamics driving these exceptional responses, thereby providing a preliminary theoretical foundation for future treatment de-escalation strategies.

## Methods

### Study population and design

**Figure 1:**
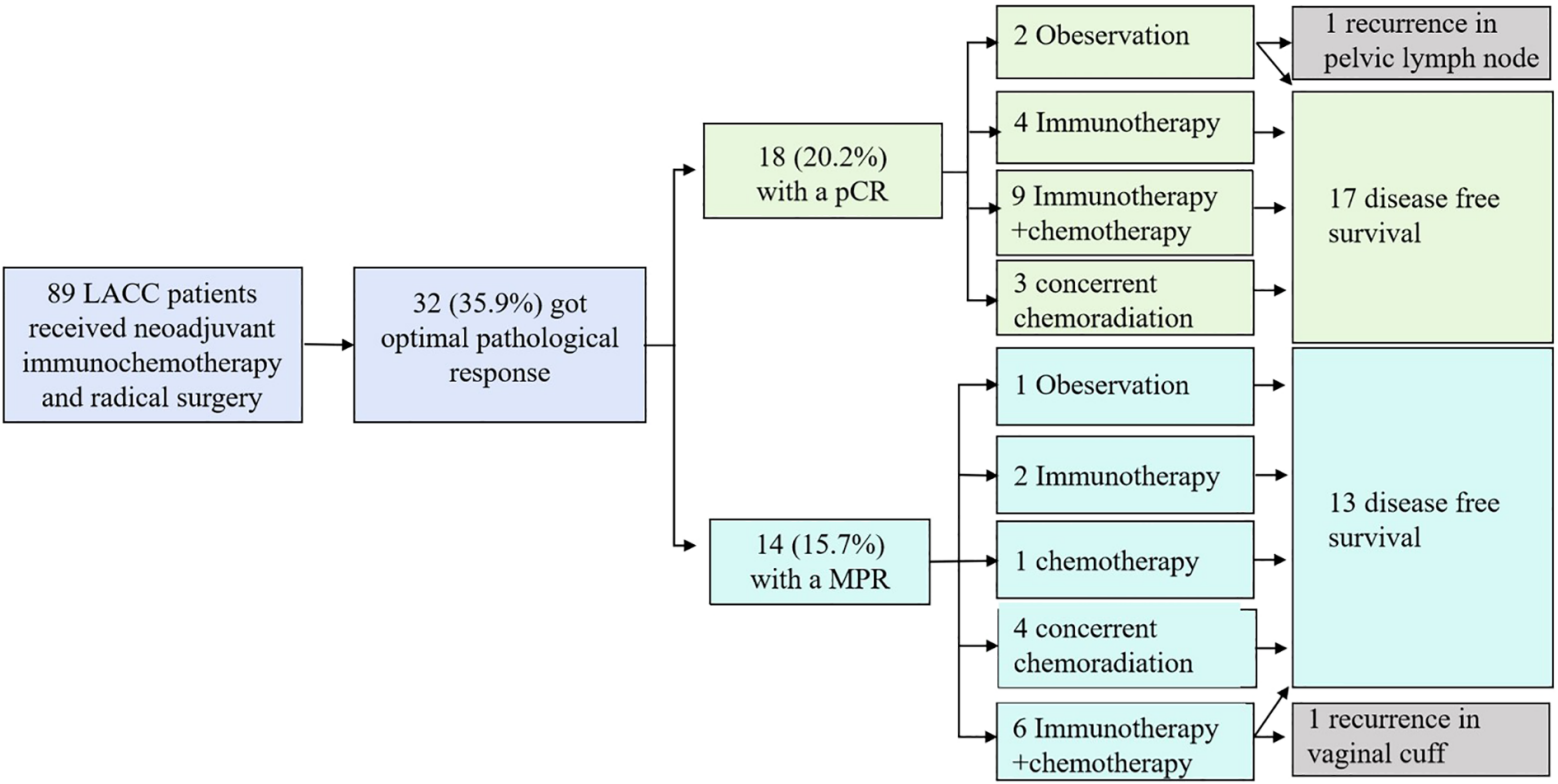
Trial profile and outcome.

A retrospective analysis was conducted on patients with LACC who received neoadjuvant chemoimmunotherapy at the Chinese PLA General Hospital (a high-volume tertiary medical system comprising multiple clinical centers) between 2022 and 2025 (**Figure 1**). The study was approved by the Ethics Committee of the Chinese PLA General Hospital (Approval No. S2024-469-02), and written informed consent was obtained from all participants. Inclusion criteria comprised: (a) receipt of neoadjuvant therapy combining immunotherapy with chemotherapy; (b) subsequent completion of radical surgery; and (c) achievement of an optimal pathological response, defined as MPR (residual tumor cells ≤10% of the original tumor volume) and or pCR (no residual tumor cells in the surgical specimen). Exclusion criteria included: (a) history of prior malignancy; (b) severe comorbidities precluding standard treatment; or (c) incomplete follow-up data.

### Data collection and definitions

Data were retrospectively collected from electronic medical records, including demographic characteristics, clinical parameters, treatment details, and safety outcomes. Specifically, we documented age, body mass index (BMI), Eastern Cooperative Oncology Group performance status (ECOG), human papillomavirus (HPV) status, gravidity, comorbidities, family history, histologic subtype, FIGO 2018 stage, neoadjuvant regimens, surgical approach, and treatment-related adverse events. Programmed death-ligand 1 (PD-L1) expression was assessed using the combined positive score (CPS), calculated as the number of PD-L1-positive cells (tumor cells, lymphocytes, and macrophages) divided by the total number of viable tumor cells, multiplied by 100.

### Treatment protocol

Tumor diameter and lymph node metastasis status were assessed using B ultrasound, computed tomography (CT), positron emission tomography-CT (PET-CT), or magnetic resonance imaging (MRI) prior to neoadjuvant therapy. The neoadjuvant chemoimmunotherapy regimens consisted of chemotherapy backbones—paclitaxel plus either cisplatin or carboplatin—combined with one of the following immune checkpoint inhibitors: tislelizumab, pembrolizumab, enlonstobart, cadonilimab, or iparomlimab and tuvonralimab. Treatment was administered in 2–3 cycles, with each cycle spaced 3–4 weeks apart.

Following neoadjuvant treatment, patients underwent radical hysterectomy with bilateral salpingo-oophorectomy and pelvic lymphadenectomy, with or without para-aortic lymphadenectomy. The surgical approach, including open, laparoscopic, or robot-assisted, was determined by the patient’s condition and surgeon preference. All laparoscopic procedures maintained low CO_2_ pneumoperitoneum pressure (10 mmHg) and avoided the use of a uterine manipulator to minimize the risk of tumor dissemination.

Postoperative adjuvant therapy was stratified based on preoperative risk factors and patient informed consent. The treatment strategies included active surveillance, immunotherapy alone, immunotherapy combined with chemotherapy, immunotherapy supplemented with radiotherapy, or concurrent chemoradiotherapy. Most patients commenced adjuvant immunotherapy within 4–8 weeks after surgery, with specific regimens individualized according to pathological findings and risk assessment. Selected patients underwent concurrent or sequential radiotherapy with total doses ranging from 45 to 50 Gy.

### Follow-up and outcomes

The primary endpoints of this study were DFS and OS. DFS was defined as the time from surgery to disease recurrence or death from any cause, while OS referred to the interval from the initiation of neoadjuvant therapy to death from any cause. Classification of treatment related adverse events followed Common Terminology Criteria for Adverse Events (CTCAE) v5.0. Patients were followed at predefined intervals through electronic medical records and telephone interviews. All participants were monitored until death, loss to follow-up, or the end of the study period (September 2025). Subgroup analyses were performed to identify potential factors influencing DFS and OS.

### Circulating tumor DNA analysis

ctDNA analysis was performed in a subset of patients using blood samples collected postoperatively following pathological diagnosis. Peripheral blood leukocytes and matched plasma samples underwent targeted sequencing with the Geneseeq Prime™ 425-gene panel (Nanjing Geneseeq Technology Inc., Nanjing, China). After duplicate read removal, mean sequencing depths of 100× for leukocytes and 5000× for plasma were achieved. Patients were classified as ctDNA-positive or ctDNA-negative based on detectable ctDNA levels.

### Transcriptomic profiling

Matched tumor tissue specimens were obtained from the identical anatomical sites in a subset of 10 patients (who achieved pCR) before neoadjuvant treatment initiation and from the corresponding surgical resection specimens. All collected samples were immediately snap-frozen in liquid nitrogen and maintained at −80°C until RNA extraction. RNA isolation and sequencing library preparation followed established protocols as previously reported (11). Differential gene expression analysis between paired pre- and post-treatment samples from 10 patients was conducted using the edgeR package (v3.22.5) in R (v4.2.1). Genes with adjusted p-values (padj) ≤ 0.05 and absolute log2 fold change ≥ 1 were defined as significantly differentially expressed genes (DEGs). Visualization of DEGs was performed using the pheatmap and ggplot2 (v3.3.6) packages. And functional annotation of the DEGs was performed by Metascape.

### Multi-algorithm immune infiltration analysis

To systematically quantify the treatment-induced remodeling of the tumor immune microenvironment (TME), a multi-algorithm consensus approach was employed. The relative abundance of tumor-infiltrating immune cells was deconvoluted from the bulk RNA sequencing data using the immunedeconv R package, which integrated six independent state-of-the-art algorithms: CIBERSORT, EPIC, MCP-counter, quanTIseq, TIMER, and xCell (12). Statistical significance between pre- and post-treatment immune cell infiltrates was assessed using the Wilcoxon matched-pairs signed-rank test, ensuring high robustness and cross-platform validation of the immunological alterations.

### Statistical analysis

All statistical analyses were conducted using R (version 4.2.1) and SPSS (version 26.0). Patient baseline characteristics were summarized using descriptive statistics, with categorical variables expressed as frequencies and percentages, and continuous variables reported as mean ± standard deviation or median with interquartile range (IQR) according to data distribution. Survival outcomes were evaluated using Kaplan-Meier methods, with between-group comparisons performed by log-rank test. A two-sided p-value below 0.05 was considered statistically significant.

## Results

### Patient baseline and characteristics

A total of 89 patients with LACC who underwent neoadjuvant chemoimmunotherapy followed by surgical resection between January 2022 and July 2025 were included in this analysis. Of these, 32 patients (35.9%) achieved a optimal pathological response, including 18 (20.2%) with a PCR and 14 (15.7%) with a MPR. The baseline clinicopathological characteristics of these 32 patients are summarized in **Table 1**. The median age was 48.5 years (IQR: 42-56.3). Regarding BMI, 10 patients (31.3%) measured below 24 kg/m², while 22 (68.8%) measured 24 kg/m² or higher. Half of the cohort (50.0%) was postmenopausal, and 12 patients (37.5%) had a gravidity of three or more. Based on preoperative imaging, maximal tumor diameter was ≤4 cm in 7 patients (21.9%), 4–5 cm in 18 (56.3%), and >5 cm in 7 (21.9%). FIGO staging distribution was as follows: IB3 in 6.2% (2/32), IIA2 in 21.9% (7/32), IIB in 25.0% (8/32), IIIC1 in 40.6% (13/32), and IIIC2 in 6.2% (2/32). Human papillomavirus (HPV) infection was detected in 29 patients (90.6%). Histologically, squamous cell carcinoma predominated (90.6%, 29/32), followed by adenocarcinoma (6.3%, 2/32) and neuroendocrine carcinoma (3.1%, 1/32). Among the 16 patients evaluated for PD-L1 expression, 14 (87.5%) exhibited a CPS ≥10. Most patients (90.6%, 29/32) had an ECOG PS of 0. None of the 17 patients tested for ctDNA showed detectable post-surgery ctDNA.

**Table 1:** Baseline characteristics in 32 patients with optimal pathological response.

Patient baseline characteristics (n = 32)	
Age in years	48.5 (42-56.3)
Body mass index (kg/m²)	24.4 (22.7-28.7)
<24	10 (31.3)
≥24	22 (68.8)
Postmenopausal	16 (50)
Gravidity	2 (1-3)
0-2	20 (62.5)
≥3	12 (37.5)
ECOG score	
0	29 (90.6)
1	3 (9.4)
Tumor size (cm)	4.4 (4.7-5.1)
≤4	7 (21.9)
>4 to ≤5	18 (56.3)
>5	7 (21.9)
FIGO stage (2018)	
IB3	2 (6.2)
IIA2	7 (21.9)
IIB	8 (25)
IIIC1	13 (40.6)
IIIC2	2 (6.2)
Histologic type	
Squamous cell carcinoma	29 (90.6)
Adenocarcinoma or neuroendocrine carcinoma	3 (9.4)
SCC (ng/mL)	3.7 (1.8-8.8)
HPV status	
16 and/or 18	20 (62.5)
Other high risk type	3 (9.4)
Negative	3 (9.4)
Not available	6 (18.8)
PD-L1 status	
CPS < 1	0
1 ≤ CPS < 10	2 (6.2)
≥10	14 (43.8)
Not available	16 (50)
ctDNA status	
Positive	0
Negative	17 (53.1)
Not available	15 (46.9)

Data are n (%) or median (IQR). ECOG, Eastern Cooperative Oncology Group performance status; FIGO, International Federation of Gynecology and Obstetrics; SCC, squamous cell carcinoma antigen; HPV, human papillomavirus; PD-L1, programmed death-ligand 1; CPS, combined positive score; ctDNA, circulating tumor DNA.

PD-L1 and ctDNA testing were performed based on clinical discretion and patient consent; the high rate of missing data reflects the real-world nature of this study and should be considered a limitation.

### Treatment strategy and adverse events

Among the 32 patients, 12 (37.5%) received three cycles of neoadjuvant immunochemotherapy, while the remaining received two or fewer cycles. The immunotherapy regimens were distributed as follows: tislelizumab was administered to 18 patients (56.3%), enlonstobart to 5 (15.6%), iparomlimab-tuvonralimab to 4 (12.5%), camrelizumab to 3 (9.4%), cadonilimab to 1 (3.1%), and pembrolizumab to 1 (3.1%). Regarding the surgical approach, 4 patients (12.5%) underwent open surgery, 19 (59.4%) laparoscopic surgery, and 9 (28.1%) robotic-assisted surgery. Among them, 26 patients additionally underwent para-aortic lymphadenectomy. One intraoperative bladder injury occurred and was successfully repaired without further complications. Four patients required blood transfusions, including three during surgery and one postoperatively. Postoperative complications are detailed as follows: seven patients (21.9%) required medical intervention, including three who received blood transfusions, two with lymphatic leakage that resolved with conservative management, one with cuff infection and urinary tract infection treated with antibiotics, one readmitted due to cuff dehiscence and bleeding which resolved after secondary suture, and one case of urinary retention requiring catheterization for two months. The grade 3 complication rate according to Clavien-Dindo Grade was 2 (6.25%), and no grade 4 was reported. The median postoperative hospital stay was 7 days (range: 5–19 days). Postoperative adjuvant treatment strategies included: no further therapy in three patients, chemotherapy combined with immunotherapy in 15, chemotherapy alone in one, maintenance immunotherapy in six, and adjuvant radiotherapy in seven. Overview of tumor response and post operative treatment strategies were screened in **Figure 1**).

Treatment-related adverse events (TRAEs) are detailed in **Table 2**. Among the 32 patients, 30 (93.8%) experienced TRAEs. The most common hematological TRAEs were anemia (81.3%) and hypoproteinemia (81.3%), while gastrointestinal reactions (40.6%) represented the most frequent non-hematological events. Seven patients (21.9%) had grade 3 TRAEs, with no grade 4 reported. Immune-related adverse events (irAEs) were observed in 4 patients (12.5%), including 2 case of hypothyroidism, once case of adrenocortical hypofunction and one case of immune-related pneumonia. All TRAEs were appropriately managed without severe sequelae.

**Table 2:** Treatment-relate adverse events in 32 patients.

TRAEs	All grade	Grade 1-2	Grade ≥3
Any TRAEs, n(%)	30 (93.8)	30 (93.8)	7 (21.9)
Anemia	26 (81.3)	23 (71.9)	3 (9.4)
Leukopenia	17 (53.1)	16 (50)	1 (3.1)
Neutropenia	16 (50)	15 (46.9)	1 (3.1)
Thrombocytopenia	6 (18.8)	4 (12.5)	2 (6.3)
Hypoproteinemia	26 (81.3)	26 (81.3)	0
Elevated liver enzymes	9 (28.1)	9 (28.1)	0
Gastrointestinal reactions	13 (40.6)	13 (40.6)	0
Skin rash	2 (6.3)	2/32 (6.3)	0
irAEs	4 (12.5)	3 (9.4)	2 (6.2)
Immune-related pneumonia	1 (3.1)	0	1 (3.1)
Adrenocortical hypofunction	1 (3.1)	0	1 (3.1)
Hypothyroidism	3 (6.2)	3 (9.4)	0

TRAEs, Treatment-relateTreatment-related adverse events; irAEs,immune-related adverse events.

### Short-term survival data

**Figure 2:**
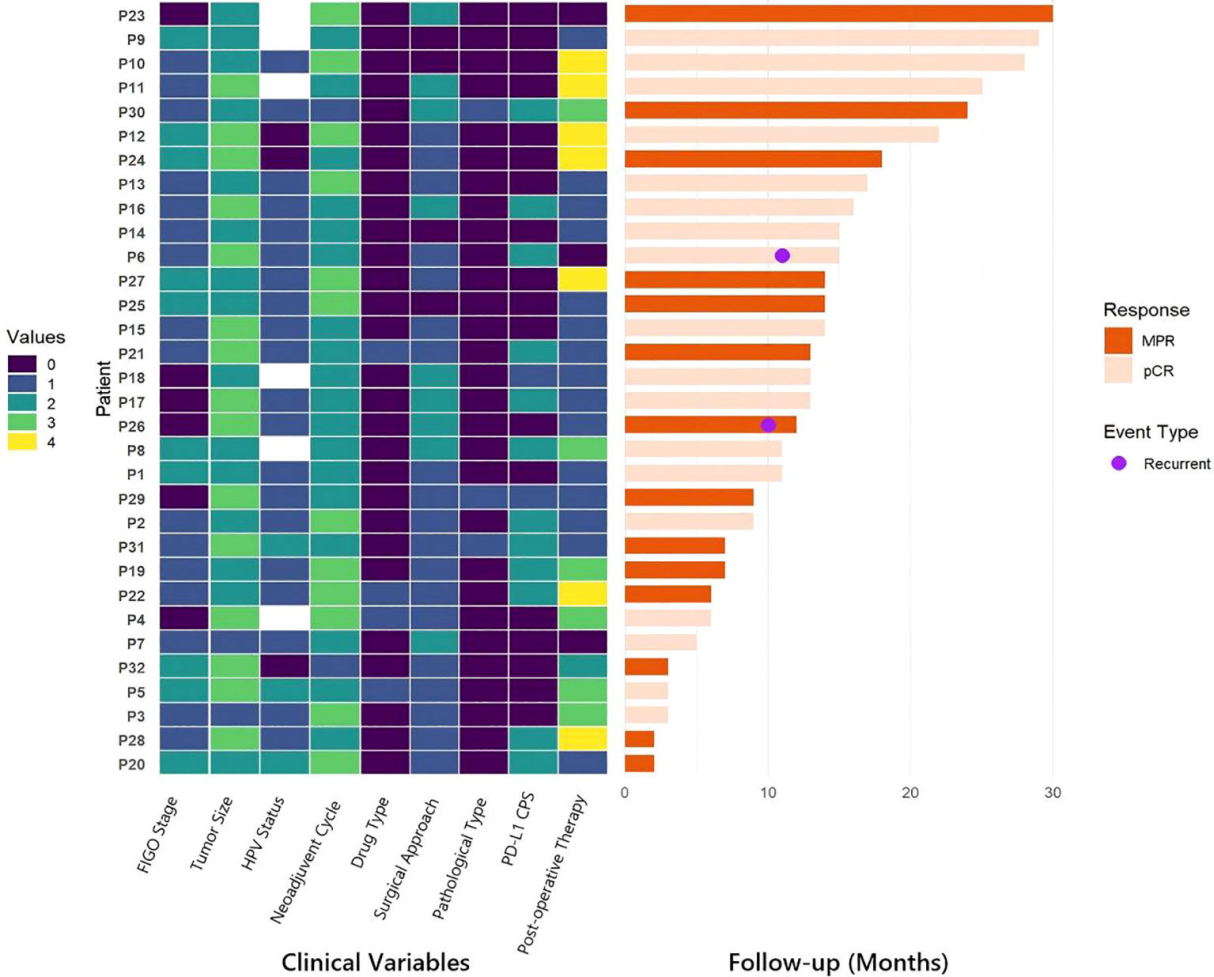
Swimmer plot of patient characteristics, treatment patterns, and pathological responses. Each horizontal bar represents an individual patient (n=32), with patient IDs shown on the left. Vertical columns display different clinical and pathological features, coded by color according to the key below. Patients are ordered by FIGO stage and pathological response status. Color Key: Tumor Size: 0=<4 cm, 1 = 4–5 cm, 2=>5 cm; FIGO Stage: 1=IB3, 2=IIA2 and IIB, 3=IIIC1 and IIIC2; HPV Status: 0=negative, 1 = 16/18 type, 2=other high-risk types; Neoadjuvant Cycles: 1 = 1 cycle, 2 = 2 cycles, 3 = 3 cycles; Drug Type: 1=anti-PD-1 monoclonal antibody, 2=anti-PD-1/CTLA-4 bispecific antibody; Surgical Approach: 0=open, 1=laparoscopic, 2=robotic; Pathological Type: 0=squamous carcinoma, 1=non-squamous carcinoma; PD-L1 CPS: 0=not available, 1=<10, 2=≥10; Postoperative Therapy: 0=observation, 1=immuno-chemotherapy, 2=chemotherapy, 3=immunotherapy, 4=radiotherapy. Abbreviations: FIGO, International Federation of Gynecology and Obstetrics; HPV, human papillomavirus; PD-L1, programmed death-ligand 1; CPS, combined positive score; pCR, pathologic complete response; MPR, major pathological response.

All patients were included in the efficacy and safety analyses. The median follow-up time for survivors was 13 months (IQR: 7-16), with a range of 3 to 31 months as of the data cut-off in September 2025; follow-up remains ongoing for all patients. Survival outcomes, pathological responses, and associated clinical characteristics are summarized in **Figure 2**. Two patients experienced recurrence during the follow-up period: one at the vaginal cuff and the other in the pelvic lymph nodes. Both patients had been preoperatively diagnosed with lymph node metastasis (stage IIIC1r) based on imaging. Notably, neither patient received postoperative radiotherapy: one declined radiotherapy and received only four cycles of postoperative chemoimmunotherapy due to her young age, while the other discontinued adjuvant therapy following grade 3 immune-related pneumonia. No deaths were observed during the follow-up period.

**Figure 3:**
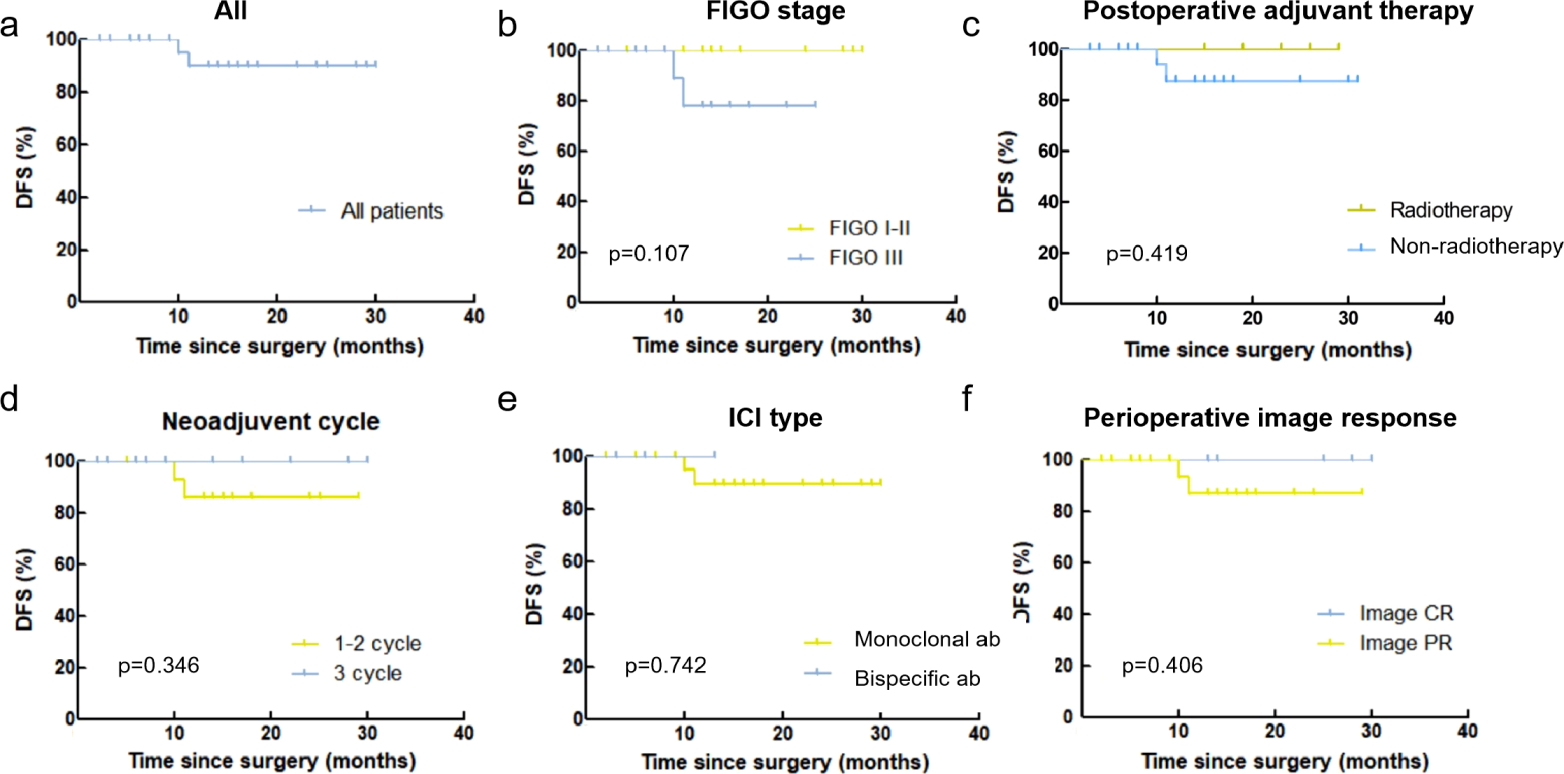
Subgroup analysis of disease-free survival in patients achieving optimal pathological response. Kaplan-Meier survival curves comparing recurrence rates stratified by potential prognostic factors. **(A)** All patients. **(B)** FIGO stage (stage I-II vs. stage III). **(C)** Postoperative adjuvant regimens (radiotherapy vs. non-radiotherapy). **(D)** Number of neoadjuvant cycles (1–2 cycles vs. 3 cycles). **(E)** Immune checkpoint inhibitor type (monoclonal antibody vs. bispecific antibody). **(F)** Imaging response before surgery (Image complete response vs. Image partial response). No statistically significant differences were observed across all subgroup comparisons (log-rank test).

To systematically evaluate the impact of treatment heterogeneity on these favorable outcomes, we categorized the neoadjuvant regimens based on both pharmacological properties and treatment intensity. Regarding the ICIs, 27 patients (84.4%) received anti-PD-1 antibodies (monoclonal antibodies), while 5 (15.6%) received anti-PD-1/CTLA-4 antibodies (bispecific antibodies). In terms of treatment duration, 20 patients (62.5%) completed 1–2 cycles of neoadjuvant therapy, and 12 (37.5%) received 3 cycles. Subsequent subgroup analyses were conducted to elucidate whether these variations influenced oncological prognosis. No significant differences in recurrence were identified based on ICI type (monoclonal antibody vs. Bispecific antibody, p=0.732), neoadjuvant cycles (1–2 cycles vs. 3 cycles, p=0.346), FIGO stage (stage I-II vs. stage III, p=0.107), postoperative adjuvant regimens (Non-Radiotherapy vs. Radiotherapy, p=0.419) or image CR before surgery (p=0.406). These findings suggest that for patients attaining OPR, the favorable short-term survival is consistently maintained across different treatment intensities and agent types. The corresponding estimated Kaplan-Meier survival curves are presented in **Figure 3**.

### Molecular characterization and functional enrichment analysis of treatment-responsive tumors

**Figure 4:**
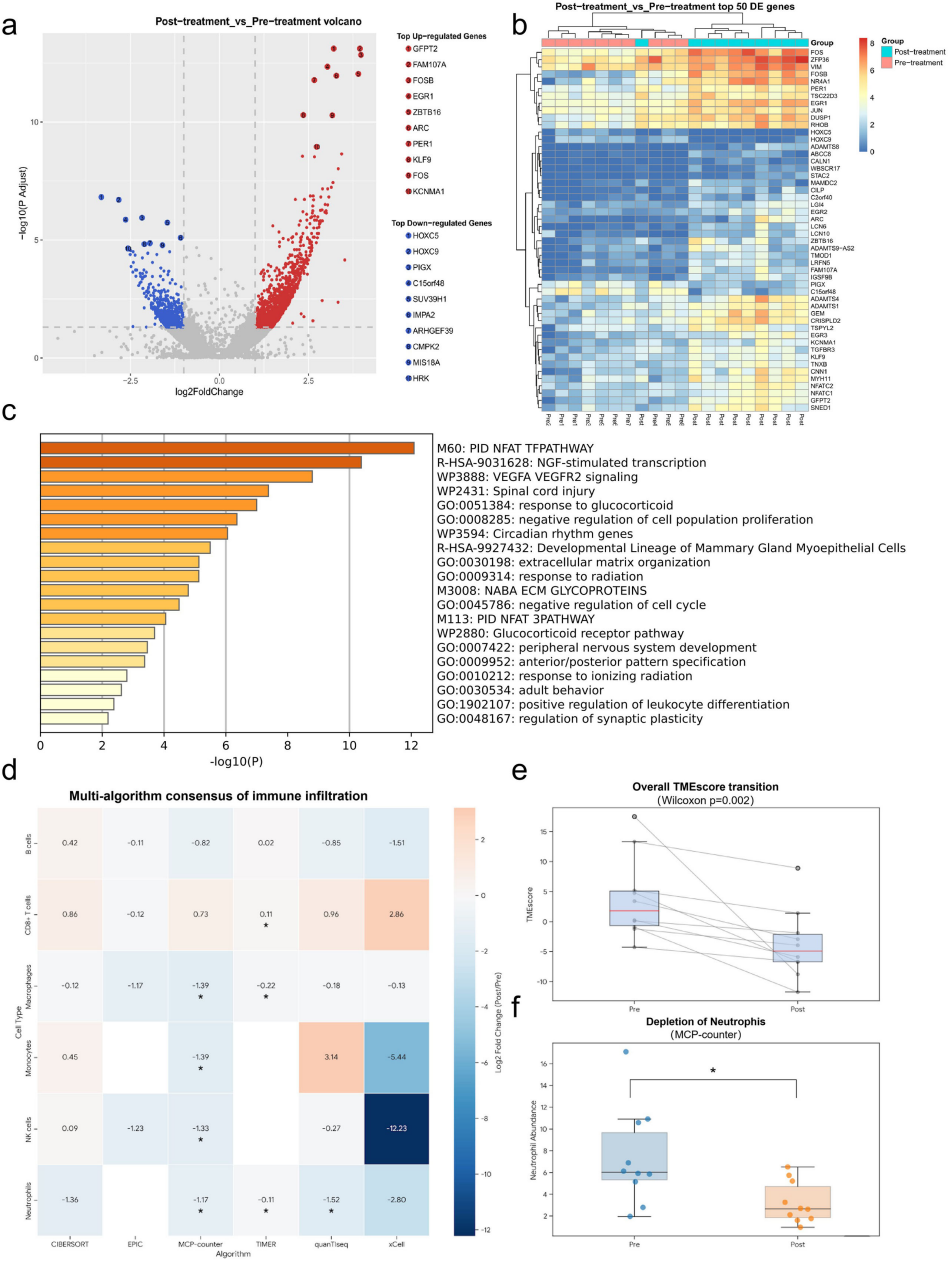
Transcriptomic landscape and tumor microenvironment remodeling in pCR patients following neoadjuvant chemoimmunotherapy. Paired pre- and post-treatment tumor specimens from 10 patients who achieved pathological complete response (pCR) were subjected to RNA sequencing. **(A)** Volcano plot displaying differentially expressed genes (DEGs) between post-treatment and pre-treatment samples. **(B)** Hierarchical clustering heatmap of the top 50 DEGs, demonstrating distinct expression patterns before and after treatment. **(C)** Pathway enrichment analysis revealing predominant biological modules associated with treatment response, including immune activation, cell cycle regulation, and extracellular matrix reorganization. **(D)** Multi-algorithm consensus immune infiltration analysis demonstrating consistent remodeling of the tumor immune microenvironment. **(E)** Box plot showing significant transition of the overall tumor microenvironment score following treatment. **(F)** Representative immune cell subset alterations, characterized by increased infiltration of cytotoxic lymphocytes and depletion of pro-tumorigenic myeloid populations (*p < 0.05).

To elucidate the underlying molecular mechanisms driving the clinical response to neoadjuvant chemoimmunotherapy, we performed transcriptomic sequencing on paired pre- and post-treatment tumor specimens from 10 patients who achieved pCR. Comparative transcriptomic analysis revealed a total of 1,717 DEGs, comprising 1,188 significantly upregulated and 529 downregulated genes in the post-treatment cohort (**Figure 4A**). The expression patterns of the top 50 DEGs, notably including the upregulation of FOSB, EGR1, and ZBTB16, are visualized in a hierarchical clustering heatmap (**Figure 4B**), underscoring a dramatic transcriptomic remodeling within the tumor tissue induced by the neoadjuvant regimen.

To further delineate the biological functions of these DEGs, we conducted pathway enrichment analysis using Metascape, revealing predominant enrichment in three clinically relevant biological modules (**Figure 4C**). First, indicating the activation of immune and inflammatory signaling, the upregulated DEGs (including FOS and FOSB) were significantly enriched in the “PID NFAT TFPATHWAY” and “positive regulation of leukocyte differentiation” (GO:1902107). Second, downregulated DEGs were strictly associated with cell cycle arrest and the suppression of tumor proliferation, featuring pathways such as “negative regulation of cell population proliferation” (GO:0008285) and “response to ionizing radiation” (GO:0010212). Finally, highlighting extracellular matrix (ECM) remodeling in the tumor bed, pathways including “extracellular matrix organization” (GO:0030198) and “NABA ECM GLYCOPROTEINS” (M3008) were heavily enriched in the post-treatment specimens.

Consistent with these transcriptomic functional alterations, our multi-algorithm immune infiltration consensus analysis (incorporating CIBERSORT, EPIC, MCP-counter, quanTIseq, TIMER, and xCell) demonstrated a profound structural reorganization of the TME. As illustrated in **Figures 4D**, OUR multi-algorithmic approach revealed a highly consistent immune remodeling landscape. Specifically, neoadjuvant chemoimmunotherapy regimen led to an overall transition of the TME score (p=0.0020, **Figure 4E**), characterized by a consistent increase in cytotoxic CD8+ T-cell infiltration and a profound depletion of pro-tumorigenic neutrophils (p<0.05 across multiple platforms, **Figure 4D, F**). This systematic cross-validation significantly strengthens our conclusion that the neoadjuvant regimen actively reverses immunosuppression, fostering a potent anti-tumor microenvironment that justifies the high pathological response rates observed.

## Discussion

This real-world study demonstrates that patients with LACC who achieved an OPR following neoadjuvant immunochemotherapy and radical surgery exhibited favorable short-term survival outcomes, with an estimated 2-year DFS rate of 90.7% and no deaths observed over a median follow-up of 13 months. Importantly, the treatment was well-tolerated with a relatively low incidence of grade ≥3 TRAEs (21.9%). These findings underscore the clinical viability of neoadjuvant chemoimmunotherapy followed by surgery as a potent therapeutic alternative for LACC.

Although CCRT remains the standard first-line treatment for LACC according to international guidelines, it is associated with reported recurrence or metastasis rates of 23.3-34.4% despite its established efficacy, along with a defined profile of long-term toxicities (13–15). Consequently, the ongoing clinical debate regarding these treatment strategies focuses on three key questions: whether surgical intervention meaningfully improves prognostic outcomes, whether postoperative adjuvant therapy leads to redundant use of medical resources, and how each strategy affects long-term quality of life (16). Our results reinforce growing evidence that neoadjuvant regimens followed by surgery represent a clinically viable alternative in real-world settings. This is particularly relevant in medical contexts with high surgical expertise and where patient preference aligns with a surgical approach (14).

Building on this surgical paradigm, the integration of ICIs represents a significant therapeutic advancement. Recent pivotal phase III trials have sought to incorporate ICIs into standard CCRT, with divergent outcomes. While the Keynote-A18 trial established a new standard by demonstrating significant PFS benefits with pembrolizumab plus CCRT (17), the CALLA trial failed to show a statistically significant PFS improvement using durvalumab plus CCRT (18). These conflicting results indicate that simply adding immunotherapy to radiotherapy does not universally guarantee success, highlighting the need for precise patient stratification. In contrast to these “radiotherapy-based” curative intents, our surgical model allows for the direct histopathological confirmation of tumor eradication. The estimated 2-year DFS of 90.7% in our OPR subgroup compares highly favorably to the overall PFS reported in these landmark trials. This suggests that achieving a deep pathological response via neoadjuvant chemoimmunotherapy successfully identifies a superior prognostic subset of patients whose survival outcomes significantly exceed the average performance of standard-of-care combinations. In parallel to CCRT-based trials, neoadjuvant chemoimmunotherapy followed by surgery has emerged as a promising frontier. A core objective of our study is to contextualize our real-world outcomes against recent prospective neoadjuvant phase II trials. The NACI study investigated neoadjuvant camrelizumab plus chemotherapy in patients with bulky LACC (stages IB3, IIA2, or IIB/IIIC1r with tumor diameter ≥4 cm), reporting a pCR rate of 38% without restrictions on immunotherapy cycle number and without MPR data reporting (8). The PACS trial, evaluating sintilimab combined with chemotherapy in 47 stage IB3-IIA2 LACC patients, demonstrated a pCR rate of 36.2% and an optimal response rate (residual tumor <3 mm) of 53.2% (10). Most notably, the NATIC study, which implemented stricter inclusion criteria and three cycles of neoadjuvant tislelizumab plus chemotherapy in stage IB3/IIA2 LACC patients, achieved remarkable results: pCR in 66.7% of patients, MPR in 13.3%, yielding an overall OPR of 80.0%. In our cohort, the overall OPR rate was 35.9%, with a strict pCR rate of 20.2%. An in-depth analysis reveals several potential factors driving these discrepancies in pCR and survival outcomes. First, baseline patient characteristics play a pivotal role; NATIC exclusively enrolled earlier-stage (IB3/IIA2) LACC with lower baseline tumor burdens, whereas our cohort and the NACI trial included more advanced cases (up to stage III/IVA). Second, treatment regimen rigor differed significantly; NATIC utilized an intensified 3-cycle regimen, whereas our patients received variable cycles (predominantly 2 cycles) of diverse ICI agents, which may have diluted the overall pCR rate. Consequently, it is crucial to clearly define the evidence level of our study: while NACI, PACS and NATIC provide high-level, prospective evidence, our study provides complementary, real-world observational evidence focusing on the post-operative trajectory and real-world feasibility of patients who have successfully bridged this treatment to achieve OPR.

Our transcriptomic and multi-algorithmic bioinformatic analyses provide a multi-dimensional perspective on how neoadjuvant chemoimmunotherapy reshapes the molecular landscape to achieve these deep responses. A pivotal finding in our pathway analysis was the robust enrichment of the NFAT (Nuclear Factor of Activated T-cells) signaling pathway and leukocyte differentiation markers. NFAT transcription factors are indispensable orchestrators of T-cell receptor (TCR)-mediated signaling, and their activation is essential for producing effector cytokines such as IFN-γ and TNF-α (19). Furthermore, the restoration of NFAT-dependent transcriptional programs is a recognized hallmark of reinvigorated exhausted T-cells during PD-1 blockade (20). The concurrent upregulation of early-response genes, specifically FOS and FOSB, further indicates that neoadjuvant chemoimmunotherapy effectively bypasses inhibitory signals within the TME, triggering a cascade of leukocyte maturation and clonal expansion (21). This molecular evidence is strongly corroborated by our consensus immune profiling, which demonstrated a significant influx of cytotoxic CD8+ T cells across all six computational algorithms. This “cold-to-hot” TME transition is a cornerstone of successful immunotherapy in solid tumors and serves as a major determinant for achieving an OPR (22). Beyond immune activation, the significant downregulation of genes associated with cell cycle progression and proliferation (e.g., SUV39H1, ARHGEF39) reflects the potent synergistic cytotoxicity of chemotherapy and PD-1 inhibition. The concurrent enrichment of “response to ionizing radiation” and “negative regulation of cell cycle” pathways suggests that the neoadjuvant regimen induces severe DNA damage and metabolic stress, ultimately leading to the senescence or apoptosis of malignant clones. The targeted downregulation of HOXC5 and HOXC9—members of the Homeobox family known to promote epithelial-mesenchymal transition and stemness in cancers (23), further suggests that neoadjuvant chemoimmunotherapy also impairs the metastatic potential of any residual tumor cells. Finally, our findings highlight a unique structural transition in the extracellular matrix (ECM) composition within the tumor bed. While ECM organization pathways are traditionally associated with tumor progression, in the context of deep pathological regression, the upregulation of specific ECM glycoproteins (e.g., GFPT2, SNED1) likely represents a “wound healing” and fibrotic repair response (24). As malignant parenchymal cells are cleared, they are replaced by fibrotic tissue and stromal remodeling. This ECM transition, coupled with the profound depletion of immunosuppressive neutrophils and macrophages, suggests that neoadjuvant chemoimmunotherapy not only eradicates tumor cells but effectively sterilizes the pro-tumorigenic niche, thereby potentially reducing the risk of long-term recurrence.

Focusing on clinical translational value, the core proposition arising from our study is the feasibility of treatment de-escalation for deep responders. Currently, the optimal postoperative management for pCR/MPR patients remains a subject of intense debate. Our findings demonstrate that while local pathological clearance is a powerful prognosticator, it must be integrated with baseline clinical risk and real-time molecular surveillance to ensure oncological safety during de-escalation. The recurrence in two IIIC1r patients despite achieving a deep pathological response at the primary site reveals that high-risk baseline characteristics can be misleading. Specifically, suspected systemic lymph node involvement may host occult micrometastases that survive neoadjuvant therapy. Similar patterns have been seen in rectal cancer, where nodal status continues to predict survival regardless of the primary tumor’s response (25). Consequently, a simplified de-escalation strategy is likely insufficient. Our study suggests a more tailored framework, drawing inspiration from successful selective adjuvant and watch-and-wait protocols established in other areas of oncology. Crucially, our data introduce post-operative ctDNA status as a pivotal “tie-breaker” in clinical decision-making. In colorectal cancer, the presence of circulating tumor DNA (ctDNA) post-surgery serves as a definitive marker of minimal residual disease, identifying patients who derive the most benefit from adjuvant therapy while sparing ctDNA-negative patients from unnecessary toxicity (26). In our cohort, the 100% post-operative ctDNA negativity among OPR patients, coupled with zero mortality, provides a compelling roadmap for biology-driven management. We hypothesize that a “dual-negative” status, defined by the convergence of major pathological response and ctDNA clearance, could serve as a validated criterion for adjuvant radiotherapy exemption. By selectively exempting these low-risk survivors from pelvic radiotherapy, we could significantly mitigate chronic gastrointestinal and urogenital morbidities, thereby preserving the quality of life without compromising the curative intent. This hypothesis-generating model warrants validation in prospective “de-intensification” trials where treatment is intensified only for those with persistent molecular or nodal risk. The integration of neoadjuvant chemoimmunotherapy with radical surgery demonstrates a manageable safety profile, effectively balancing therapeutic potency with clinical feasibility. While TRAEs were frequent, they were predominantly controllable and did not compromise the overall treatment course. Consistent with the known toxicity profiles of platinum-based regimens,hematological toxicities, specifically anemia and hypoproteinemia (each 81.3%), were the most prevalent, whereas gastrointestinal reactions (40.6%) constituted the primary non-hematological toxicity. Importantly, the incidence of grade 3 TRAEs was limited to 21.9%, with no grade 4 events or treatment-related deaths recorded. The incidence of irAEs was relatively infrequent (12.5%)and primarily manifested as endocrine disorders that were readily managed with standard protocols. From a surgical perspective, the complication profile aligned with expectations for radical gynecologic oncology procedures. Although 21.9% of patients required medical intervention for postoperative complications, only two (6.25%) experienced Clavien-Dindo grade 3 events. The surgical challenge may be partially attributed to tissue changes induced by neoadjuvant therapy, which can alter dissection planes and increase operative difficulty. However, all complications-including one intraoperative bladder injury successfully repaired without sequelae, two cases of lymphatic leakage managed conservatively, and one case of cuff dehiscence requiring readmission and secondary suture-were promptly identified and effectively addressed through standardized perioperative protocols. Collectively, these safety observations support the feasibility of incorporating immunotherapy into the multimodal management of LACC without introducing unmanageable toxicities. Several core limitations must be rigorously articulated in interpreting our results. First, the retrospective, non-randomized design inherently carries the risk of selection bias. Second, the limited statistical power resulting from the small sample size and relatively short follow-up duration restricts long-term conclusions; thus, the estimated 2-year DFS must be interpreted with caution. Third, the clinical heterogeneity introduced by varied immunotherapy agents and regimens complicates the isolation of specific drug efficacies. Fourth, the absence of a non-responder group or concurrent CCRT control group prevents us from directly quantifying the incremental benefit of neoadjuvant chemoimmunotherapy over the current standard of care. Finally, regarding our molecular investigations, the evidence level remains preliminary. The transcriptomic analysis was performed on an exploratory subset of patients, and the conclusions are strictly hypothesis-generating. The high rate of missing data for key biomarkers, such as PD-L1 expression (50% missing) and ctDNA status (46.9% missing), further limits our ability to establish robust correlations. Future large-scale, prospective multicenter trials with standardized protocols and extended follow-up are needed to validate these findings.

## Conclusions

This study demonstrates that LACC patients achieving pCR or MPR after neoadjuvant chemoimmunotherapy exhibit excellent short-term outcomes and a manageable safety profile. Our multi-dimensional transcriptomic analyses reveal that these optimal responses are driven by profound immune reinvigoration, including NFAT pathway activation and a “cold-to-hot” microenvironmental transition. Further prospective validation in larger cohorts is warranted to standardize the management of this highly responsive subgroup.

## Data Availability

The datasets presented in this study can be found in online repositories. The names of the repository/repositories and accession number(s) can be found in the article/supplementary material.
